# Microglial activation by microbial neuraminidase through TLR2 and TLR4 receptors

**DOI:** 10.1186/s12974-019-1643-9

**Published:** 2019-12-02

**Authors:** María del Mar Fernández-Arjona, Jesús M. Grondona, Pedro Fernández-Llebrez, María Dolores López-Ávalos

**Affiliations:** 1 Dpto. de Biología Celular, Genética y Fisiología, Facultad de Ciencias, Universidad de Málaga, Campus de Teatinos, 29071 Málaga, Spain; 2 Instituto de Investigación Biomédica de Málaga-IBIMA, Málaga, Spain

**Keywords:** TLR2, TLR4, Neuraminidase, Microglia, Neuroinflammation, Oseltamivir, N-acetyl-2,3-dehydro-2-deoxyneuraminic acid

## Abstract

**Background:**

Neuraminidase (NA) is a sialidase present, among various locations, in the envelope/membrane of some bacteria/viruses (e.g., influenza virus), and is involved in infectiveness and/or dispersion. The administration of NA within the brain lateral ventricle represents a model of acute sterile inflammation. The relevance of the Toll-like receptors TLR2 and TLR4 (particularly those in microglial cells) in such process was investigated.

**Methods:**

Mouse strains deficient in either TLR2 (TLR2^-/-^) or TLR4 (TLR4^-/-^) were used. NA was injected in the lateral ventricle, and the inflammatory reaction was studied by immunohistochemistry (IBA1 and IL-1β) and qPCR (cytokine response). Also, microglia was isolated from those strains and in vitro stimulated with NA, or with TLR2/TLR4 agonists as positive controls (P3C and LPS respectively). The relevance of the sialidase activity of NA was investigated by stimulating microglia with heat-inactivated NA, or with native NA in the presence of sialidase inhibitors (oseltamivir phosphate and N-acetyl-2,3-dehydro-2-deoxyneuraminic acid).

**Results:**

In septofimbria and hypothalamus, IBA1-positive and IL-1β-positive cell counts increased after NA injection in wild type (WT) mice. In TLR4^-/-^ mice, such increases were largely abolished, while were only slightly diminished in TLR2^-/-^ mice. Similarly, the NA-induced expression of IL-1β, TNFα, and IL-6 was completely blocked in TLR4^-/-^ mice, and only partially reduced in TLR2^-/-^ mice. In isolated cultured microglia, NA induced a cytokine response (IL-1β, TNFα, and IL-6) in WT microglia, but was unable to do so in TLR4^-/-^ microglia; TLR2 deficiency partially affected the NA-induced microglial response. When WT microglia was exposed in vitro to heat-inactivated NA or to native NA along with sialidase inhibitors, the NA-induced microglia activation was almost completely abrogated.

**Conclusions:**

NA is able to directly activate microglial cells, and it does so mostly acting through the TLR4 receptor, while TLR2 has a secondary role. Accordingly, the inflammatory reaction induced by NA in vivo is partially dependent on TLR2, while TLR4 plays a crucial role. Also, the sialidase activity of NA is critical for microglial activation. These results highlight the relevance of microbial NA in the neuroinflammation provoked by NA-bearing pathogens and the possibility of targeting its sialidase activity to ameliorate its impact.

## Introduction

Toll-like receptors (TLRs) are a large family of membrane proteins which recognizes conserved structural motifs found in microbes, the so-called *pathogen-associated molecular patterns* (PAMPs), as well as patterns belonging to the individual itself, the *damage-associated molecular patterns* (DAMPs) [1, 2]. Therefore, they serve as sensitive sensors and contribute to a first line of defense in the immune response. TLRs are widespread but are particularly expressed in immune cells. Among the various subtypes described so far, TLR2 and TLR4 are relevant (although not the only) in the initiation of the inflammatory response within the central nervous system (CNS) [3–5].

In bacterial and viral CNS infections, the participation of receptors TLR2 and TLR4 has been documented [5, 6]. Peptidoglycans of bacterial cell wall, and lipopolysaccharide (LPS) of the outer membrane of Gram-negative bacteria, are well known specific ligands of TLR2 and TLR4 respectively. Both trigger intracellular signaling pathways, specifically a MyD88-dependent pathway that ends in the activation of the nuclear factor kappa B (NF-κB), with the subsequent increase in the expression of pro-inflammatory cytokines such as interleukin 1 beta ( IL-1β) and tumor necrosis factor alpha (TNFα) [3, 5, 7]. TLR4 signaling can also elicit the synthesis of interferon beta (IFNβ) [8]. Besides, the activation of TLR2 and TLR4 results in the increased production of reactive oxygen species (ROS), which contributes to pathogen death and to T-lymphocyte activation [9].

Apart from peptidoglycans and LPS, other known microbial PAMPs include glycoproteins, glycolipids and mannose-rich glycans, flagellin, porins, lipoteichoic acids, various microbial lipids, zymosan, and bacterial and viral nucleic acids (like single- or double-stranded viral RNA) [10–15]. The knowledge about microbial components recognized as PAMPs is valuable in the design of strategies (e.g., vaccines) to fight infections.

The enzyme neuraminidase (NA) is part of the envelope of certain viruses, among which is influenza virus [16]. It can also be found in a wide range of bacteria, both pathogenic or not [17, 18]. Some of these NA-bearing microbes may invade the CNS and produce infections, as is the case of mumps virus [19], several strains of bacteria-producing meningitis [20], and occasionally even *Clostridium perfringens* [21]. Besides, reports of influenza virus provoked neurologic complications are frequent [22–24]. In some of these pathogens, NA has been related to the infection and/or dispersion mechanisms, and therefore to their virulence, as is the case of influenza virus [25]. In fact, some of the most extended treatments for flu infection consist of NA inhibitors such as oseltamivir [26].

The importance of NA in the CNS infective process provoked by these pathogens can be modeled by its intracerebroventricular (ICV) administration in experimental animals [27, 28]. The sole presence of NA within the ventricular system triggers an acute neuroinflammatory process, evidenced by cellular infiltration, gliosis, and pro-inflammatory cytokines expression [29, 30]. Also, NA itself can induce the activation of the complement system, which is present in the cerebrospinal fluid [31]. Other damages provoked by NA in the CNS include myelin vacuolation, ependymal denudation, ventriculomegaly, and eventually hydrocephalus [27, 28, 32]. The wide range of effects of NA within the CNS makes it worth to explore its mechanism of action. For example, it is unknown if TLRs are involved in the inflammatory process induced by NA and if these receptors recognize NA as a PAMP. To investigate this, we applied this NA-induced inflammation model to wild type (WT), TLR2 knockout (TLR2^-/-^), and TLR4 knockout (TLR4^-/-^) mice, and evaluated the extent of the inflammatory process.

Microglial cells represent the first line of defense against pathogens invading the CNS, and they express several isoforms of TLRs [4–6]. To explore the relevance of TLR signaling in microglial cells during NA-induced inflammation, microglia cultures obtained from WT, TLR2^-/-^, and TLR4^-/-^ mice were in vitro stimulated with NA. Finally, as NA has a sialidase activity, the relevance of such activity for the effects of NA on microglia was investigated as well.

## Material and methods

### Animals

TLR2 (B6.129-Tlr2^tm1kir^/J) and TLR4 (B6.B10ScN-Tlr4^lps-del^/JthJ)-deficient mice (TLR2^-/-^ and TLR4^-/-^ respectively) were breed by The Jackson Laboratory and purchased through Charles River Laboratories (Lyon, France). The wild-type strain used as control was C57BL/6 J. These animals were maintained in the animal house at Universidad de Málaga, under a 12 h light/dark cycle, at 23 °C and 60% humidity, with food and water available ad libitum. Animal care and handling were performed according to the guidelines established by Spanish legislation (RD 53/2013) and the European Union regulation (2010/63/EU). All procedures performed were approved by the ethics committee of Universidad de Málaga (Comité Ético de Experimentación de la Universidad de Málaga; reference 2012-0013). All efforts were made to minimize the number of animals used and their suffering.

### Intracerebroventricular injection

An acute and sterile neuroinflammatory process was generated in mice by a single injection of the enzyme NA within the right lateral ventricle of the brain [27, 28]. Sham rodents were injected with 0.9% sterile saline. Animals were sacrificed at different times after the injection. Intracerebroventricular (ICV) injection procedure was performed as previously described in rats [29], but in this case, fine-tuned for mice. Briefly, the animals were anesthetized and positioned in a stereotaxic frame. A scalp incision along the sagittal midline was performed to access the skull and the bone was perforated with a drill in the following coordinates: 0.1 mm posterior and 0.9 mm lateral from Bregma [33]**.** NA from *Clostridium perfringens* (Sigma-Aldrich, N3001) dissolved in 0.9% sterile saline was administered by a single injection 2.0 mm below the dura mater with the aid of a pump; the amount of NA injected was 75 mU in 1 μL, perfused during 5 min at a rate of 0.2 μL/min. The animals were sacrificed at different times post-injection and their brains were used for RNA extraction or for immunohistochemistry (6 and 24 h, respectively).

### Isolation and culture of microglial cells

Microglial cells were isolated according to Saura’s method [34]. The mix cell cultures were obtained from 3 to 5-day-old mice sacrificed by decapitation. The brains were dissected out and meninges eliminated. Cortical brain tissue was disrupted in the presence of 0.25% trypsin solution plus 1 mM of ethylenediaminetetraacetic acid (EDTA) in Hank’s Balanced Salt Solution (HBSS; Sigma). After mechanical and chemical dissociation, cells were harvested by centrifugation (300*×g*, 10 min) and seeded in Dulbecco’s Modified Eagle Medium/Nutrient Mixture F-12 (DMEM-F12; Gibco), supplemented with 10% fetal bovine serum (FBS, Sigma-Aldrich) and 1% penicillin/streptomycin (Sigma-Aldrich) at a density of 250,000 cells/mL, and cultured at 37 °C in humidified 5% CO_2_. The medium was replaced every 5 days; confluence was achieved after 2 weeks. After maintaining the culture in confluence for 1–2 weeks, microglial cells were isolated by the following mild trypsinization method. The medium was removed and the mixed culture carefully washed; then it was treated with 0.25% trypsin solution plus 1 mM EDTA in HBSS during 1 h at 37 °C. During this incubation, the main monolayer detaches like a sheet, leaving exposed the microglia underneath which is adhered to the plate bottom. The purity of these microglial cultures was checked by immunocytochemistry and usually was about 95%. The average yield with this method was about 5000 cells/well in 24 multiwell plates and about 20,000 cells/well in 12 multiwell plates (Sigma-Aldrich, TPP tissue culture plates). Microglial cultures were obtained from wild type, TLR2^-/-^ and TLR4^-/-^ mouse strains. When required, microglial cultures were activated by the addition of [1] the synthetic triacylated lipoprotein Pam3CSK4 (P3C; InvivoGen, 12A10-MM; 2 μg/mL), which is an agonist of TLR2 [2]; ultrapure (a highly purified fraction from *E. coli*) lipopolysaccharide (LPS; InvivoGen, 13I06-MM; 5 μg/mL), which is a specific ligand of TLR4; or [3] NA (Sigma-Aldrich, N3001; 50 mU/mL), whose effect on microglial cells is under investigation herein.

### Sialidase activity inactivation and inhibition

The sialidase activity of NA was eliminated by either a heat treatment or with inhibitors. For heat-inactivation, NA (50 mU/mL) was incubated at 60 °C during 20 min. The sialidase inhibitors used were oseltamivir phosphate (Sigma-Aldrich, 100 μg/mL) or N-acetyl-2,3-dehydro-2-deoxyneuraminic acid (NADNA; Sigma-Aldrich, 250 μg/mL).

To verify the inactivation or inhibition of the sialidase activity of NA, lectin histochemistry was performed on tissue sections. Briefly, deparaffinized brain sections were treated with native NA, or with heat-inactivated or inhibited NA, for 24 h at 37 °C. The sialidase activity of NA was evaluated by the removal of sialic from the ependymal surface. The presence of sialic acid was evidenced by the biotinylated lectin *Sambucus nigra* agglutinin (SNA, Vector Laboratories), chosen for its affinity to sialic acid residues. The tissue sections were incubated with SNA (10 μg/mL) diluted in PBS, for 1 h at 37 °C. The biotinylated lectin bound to the tissue was later identified by the ABC system (see “Immunohistochemistry” section below).

The inactivated/inhibited NA was used to stimulate cultured microglial cells. Native or inactivated/inhibited NA was added to microglial cells isolated from WT mice and was kept for 24 h. Microglia were then harvested for RNA isolation and quantitative PCR (qPCR). The stimulation was evaluated by the expression of the cytokines IL-1β and TNFα.

### Immunohistochemistry

Free-floating vibratome sections were first treated, to quench endogenous peroxidase, with 10% methanol and 3% hydrogen peroxide in PBS during 45 min. After washings with PBS, nonspecific binding sites were saturated with PBT solution (0.3 % bovine serum albumin, 0.3% Triton X-100 in PBS pH 7.3). Primary antibodies were rabbit anti-rat IBA1 (Wako), and goat anti-rat IL-1β (R&D Systems), both diluted 1:500 in PBT solution. The primary antibodies were incubated overnight at 4 °C. The following morning the sections were washed with PBS and incubated with the appropriate biotinylated secondary antibody (goat anti-rabbit, Pierce; or rabbit anti-goat, Vector) diluted 1:1000 in PBT, at room temperature for 1.5 h. The avidin-biotin-complex amplification system (ABC; 1:250 dilution; Thermo Fisher Scientific) was used afterwards (at room temperature, 45 min) to detect the secondary biotinylated antibodies. The peroxidase activity was revealed with 0.05% diaminobenzidine and 0.03% hydrogen peroxide in PBS for 10 min. After thorough washes, the sections were then mounted onto gelatin-coated slides, air-dried, dehydrated in graded ethanol, cleared in xylene, and coverslipped with Eukitt mounting medium.

### RNA isolation

Animals were anesthetized and transcardially perfused with heparinized 0.9% saline. The brains were then extracted and properly dissected under RNase-free conditions. Immediately after dissection, tissue samples were immersed in RNA later (Sigma-Aldrich) and kept overnight at 4 °C. The next day, this solution was removed and the tissue pieces placed at – 80 °C for storage until RNA isolation.

Total RNA from the hypothalamic region was isolated using TRIzol reagent (Invitrogen). One milliliter of reagent was added to about 100 mg of tissue, and RNA was isolated following manufacturer’s instructions. Finally, the RNA was resuspended in a volume of ≤ 50 μL of RNase-free water.

When RNA was isolated from microglia cultures, the culture medium was removed, the cells washed with cold PBS, and TRIzol reagent was added to the well (0.5–1 mL, depending on the type of culture plate), to have about 20 × 10^3^ cells/mL of TRIzol. Cells were homogenized by passing TRIzol solution through a pipette several times. RNA isolation proceeded as indicated by the manufacturer, adjusting the volume of reagents when required (0.5 mL starting volume of TRIzol).

The concentration of RNA was measured in a NanoDrop microvolume spectrophotometer (NanoDrop 1000, Thermo Fisher Scientific). The A_260/280_ ratio of the isolated RNA was usually about 1.8.

### Reverse transcription

Before preparing the reverse transcription (RT) reaction, RNA samples were diluted with RNase-free water in order to bring them to a similar range of RNA concentration. cDNA synthesis from isolated total RNA was performed using the SuperScript^TM^ III First-Strand Synthesis (Invitrogen). The reaction mix was prepared according to the manufacturer’s protocol, and RNA was added to have ≈ 500 ng in a final volume of 20 μL. The RT reaction was carried out in a thermocycler (MasterCycler Gradient, Eppendorf). In these conditions, the resulting cDNA concentration should be equivalent to 25 ng RNA/μL. cDNA was stored at – 20 °C

### Quantitative PCR

Real-time PCR was used to quantify specific mRNAs represented in cDNA samples. The hot start reaction mix FastStart Essential DNA Green Master (Roche), based on SYBR Green I fluorescence dye, was used for this purpose. PCR reactions were prepared following manufacturer’s instructions. Forward and reverse primers were used at a final concentration of 0.4 μM and cDNA at 10 ng (amount established after preliminary trials). The qPCR reaction was carried out in a LightCycler 96 Instrument (Roche). The information obtained (amplification curves, melting curves and crossing points, CP, or cycle threshold, Ct) for each transcript was processed using the software provided with the LightCycler equipment.

To estimate the PCR efficiency (*E*), serial dilutions of the cDNA samples were amplified, and *E* is calculated according to the equation *E* = 10^[-1/slope]^ [35]. Relative quantification was based on the level of expression of a target gene relative to the level of expression of a reference gene [36], which was glyceraldehyde 3-phosphate dehydrogenase (GAPDH).

Primers to target the mRNA of genes related to inflammation (Table 1) were designed using the program Primer3 (https://primer3.org/). Target genes sequences were obtained from the Genbank NCBI Reference Sequence (https://www.ncbi.nlm.nih.gov/).

**Table 1:** Sequence of primers used for qPCR

Gene	Primers (5′→3′)	Product size (bp)	Accession #
GAPDH	TGAACGGGAAGCTCACTGG	307	NM_008084.3
	TCCACCACCCTGTTGCTGTA		
IL-1β	GAGTGTGGATCCCAAGCAAT	201	NM_008361.4
	ACGGATTCCATGGTGAAGTC		
TNFα	GATTATGGCTCAGGGTCCAA	197	NM_013693.3
	ACAGAGGCAACCTGACCACT		
IL-6	TTCCATCCAGTTGCCTTCTT	199	NM_031168.2
	CAGAATTGCCATTGCACAAC		

### Image acquisition

Images of immunostained tissue were acquired for cell quantification purposes; sometimes cells were counted directly under the microscope. Photographs were systematically taken from the brain areas of interest, using a digital camera (Nikon Digital Sight, DS.Fi1) coupled to an optical microscope (Leica, model DMLB) using NIS Elements software F2.20 (Nikon).

### Statistical analysis

Comparisons of data were carried out using SPSS Statistics software. The Kolmogorov-Smirnov normality tests, along with the Levene homoscedasticity test, were used to verify if data could be analyzed by parametric methods. Two-way analysis of variance (ANOVA) was used to compare groups’ means of immunopositive-cell counts or qPCR gene expression. The Kruskal-Wallis test was performed for the non-parametric data. Pairwise multiple comparisons were performed by Tukey’s or Bonferroni tests. Non-parametric data was analyzed by pairwise comparisons done with Holm-Sidak or Tamhane tests. Differences were considered significant when a *P* value < 0.05 was obtained.

## Results

### Intracerebroventricular NA increased the number of IBA1-positive and IL-1β-positive cells and induced the overexpression of pro-inflammatory cytokines in a TLR2/TLR4-dependent manner

Wild-type, TLR2^-/-^ and TLR4^-/-^ mice were ICV injected with NA, and sacrificed 24 h later. Brain sections were subjected to immunolabeling with selected markers: i) IBA1, which labels microglia and peripheral, perivascular, and meningeal macrophages; ii) IL-1β, a pro-inflammatory cytokine expressed in microglia which indicates its pro-inflammatory activation.

**Fig. 1:**
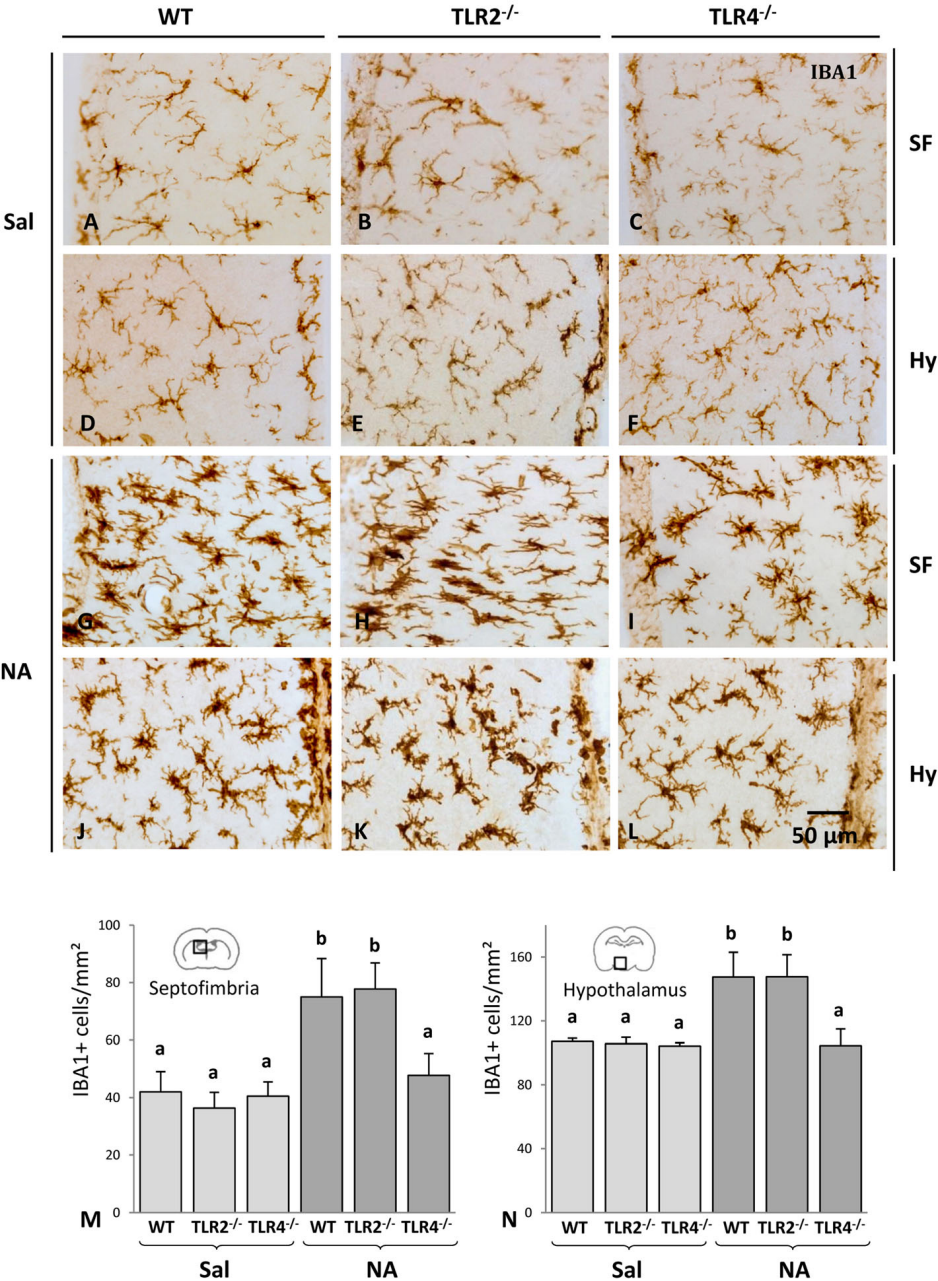
IBA1 immunostaining and cell counts after NA-induced neuroinflammation in TLR2- or TLR4-deficient mice. Animals were ICV injected with saline or NA and sacrificed 24 h after the injection. The microglia located in the septofimbria (**a**–**c**, **g**–**i**) and the hypothalamus (**d**–**f**, **j**–**l**) was analyzed. Upon NA-injection, IBA1 staining of microglial cells increased in WT as well as in TLR2^-/-^ and TLR4^-/-^ mice (**g**–**l**). Besides, cells presented a less ramified morphology, except for those from TLR4^-/-^ mice, where microglia appeared more ramified than that of WT or TLR2^-/-^ mice. IBA1-positive cell counts (**m**–**n**) revealed an increase in microglial cells after NA-injection both in the septofimbria (**m**) and in the hypothalamus (**n**). However, such increase did not occur in the TLR4^-/-^ strain, which presented IBA1-positive cell counts similar to those of the saline-treated controls. The histograms present the mean + SD of *n* = 3 saline injected or *n* = 9–10 NA-injected animals (for each strain). The Kruskal-Wallis test was used for comparing group means. Letters a and b above bars indicate the absence (if the same letter appears) or presence (if different letters appear) of a statistical difference between groups (*P* < 0.001). SF = septofimbria; H = hypothalamus; Sal = saline treatment; NA = neuraminidase treatment

Mice injected with saline showed a homogeneous distribution of microglial cells in the septofimbria (Fig. 1a–c) and the hypothalamus (Fig. 1d–f). Microglia presented a moderate IBA1 staining and a ramified morphology. No apparent differences were observed between saline-injected WT (Fig. 1a, d), TLR2^-/-^ (Fig. 1b, e), and TLR4^-/-^ (Fig. 1c, f) mice.

IBA1 staining considerably increased after NA injection in the three strains, both in the septofimbria (Fig. 1g–i) and in the hypothalamus (Fig. 1j–l), indicating the activation of microglial cells. Not only IBA1 staining increased but also cells appeared partially de-ramified and with a larger cell body. This morphological change was more evident in the septofimbria (compare Fig. 1a and g) than in the hypothalamus (compare Fig. 1d and j). The morphological change of microglia induced by NA has been previously reported in both brain regions [30]. Both TLR2^-/-^ mice (Fig. 1h, k) and TLR4^-/-^ mice (Fig. 1i, l) showed a similar response to NA to that observed in WT mice (Fig. 1g, j), that is, IBA1 overexpression, enlargement of the cell body, and some thickening and shortening of ramifications.

IBA1-positive cell counts were performed in the septofimbria (close to the ICV injection site; Fig. 1m) and in the hypothalamus (farther form the injection site; Fig. 1n). In both locations, the number of IBA1-positive cells increased after NA-injection compared to saline-injected animals. However, such increase occurred only in WT and TLR2^-/-^ animals (*P* < 0.001 vs corresponding saline), but not in TLR4^-/-^ mice, where cell counts were similar to those of the saline-treated group.

**Fig. 2:**
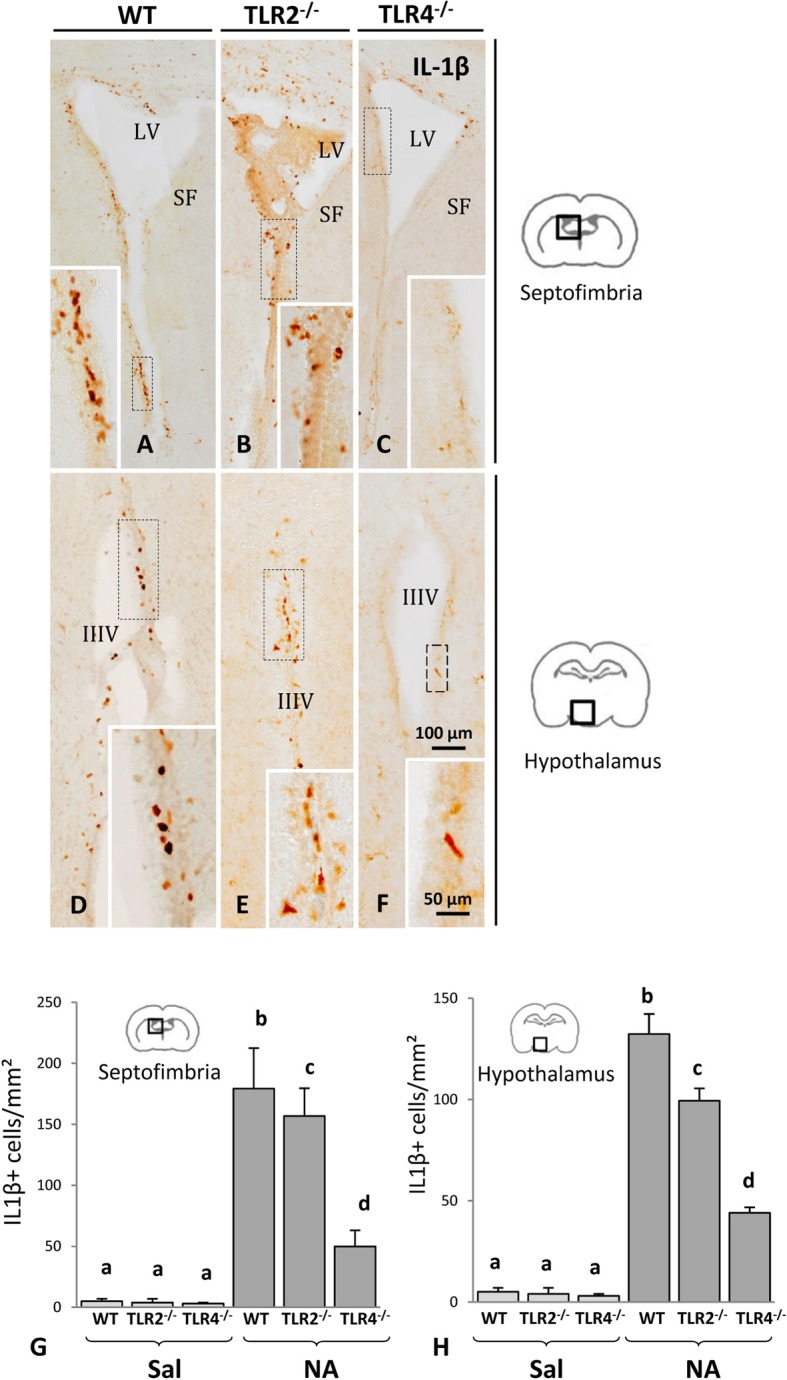
IL-1β immunostaining and cell counts after NA-induced neuroinflammation in TLR2- or TLR4-deficient mice. Histological images from septofimbria (**a**–**c**) and hypothalamus (**d**–**f**) of WT (**a**, **d**), TLR2^-/-^ (**b**, **e**), and TLR4^-/-^ (**c**, **f**) strains 24 h after the injection of NA. Staining was evident in subventricular cells or in cells close to the ventricular surface, and decreased towards deeper regions in the nervous parenchyma. Positive IL-1β label was almost undetectable in samples treated with saline (not shown). Within each image, a magnified detail is shown. IL-1β positive cells were counted in the septofimbria (**g**) and the hypothalamus (**h**). In all three strains, NA provoked an increase in IL-1β positive cells, although such increase was slightly lower in TLR2^-/-^, and significantly reduced in TLR4^-/-^, compared to WT mice. Bars in histograms are the mean + SD of *n* = 3 saline injected or *n* = 9–10 NA-injected animals (for each strain). Kruskal-Wallis test was used to compare group means. Letters (a–d) on top of the bars indicate the absence (if same letter) or presence (if different letters) of a significant difference between the compared groups (*P* < 0.001). LV = lateral ventricle; SF = septofimbria; IIIV = third ventricle

In microglial cells, IL-1β expression is associated with a proinflammatory state. This cytokine is overexpressed in microglial cells few hours after NA stimulation [30]. The number of IL-1β positive cells provides valuable information about the inflammation degree coming about in the tissue. While saline-treated animals were essentially devoid of IL-1β positive cells (not shown) 24 h after ICV injection, NA administration resulted in the emergence of IL-1β positive cells in various brain locations, particularly in periventricular areas including the septofimbria (Fig. 2a–c) and the hypothalamus (Fig. 2d–f). The intensity of the IL-1β immunostaining was higher in WT mice (Fig. 2a, d), more moderate in TLR2^-/-^ mice (Fig. 2b, e), and quite faint in TLR4^-/-^ mice (Fig. 2c, f). These observations were supported by IL-1β-positive cell counts, as NA injection resulted in increased IL-1β cell counts in the three strains when compared with their respective saline controls (Fig. 2g–h). While in WT mice, NA provoked a remarkable increase in the number of IL-1β-positive cells (*P* < 0.001 vs saline WT), a similar although milder increase was observed in TLR2^-/-^ mice (*P* < 0.001 vs NA WT). In TLR4^-/-^ mice, NA also induced expression of IL-1β in some cells, but in this case in a more restricted population than in WT or TLR2^-/-^ (*P* < 0.001 vs NA WT or NA TLR2^-/-^).

**Fig. 3:**
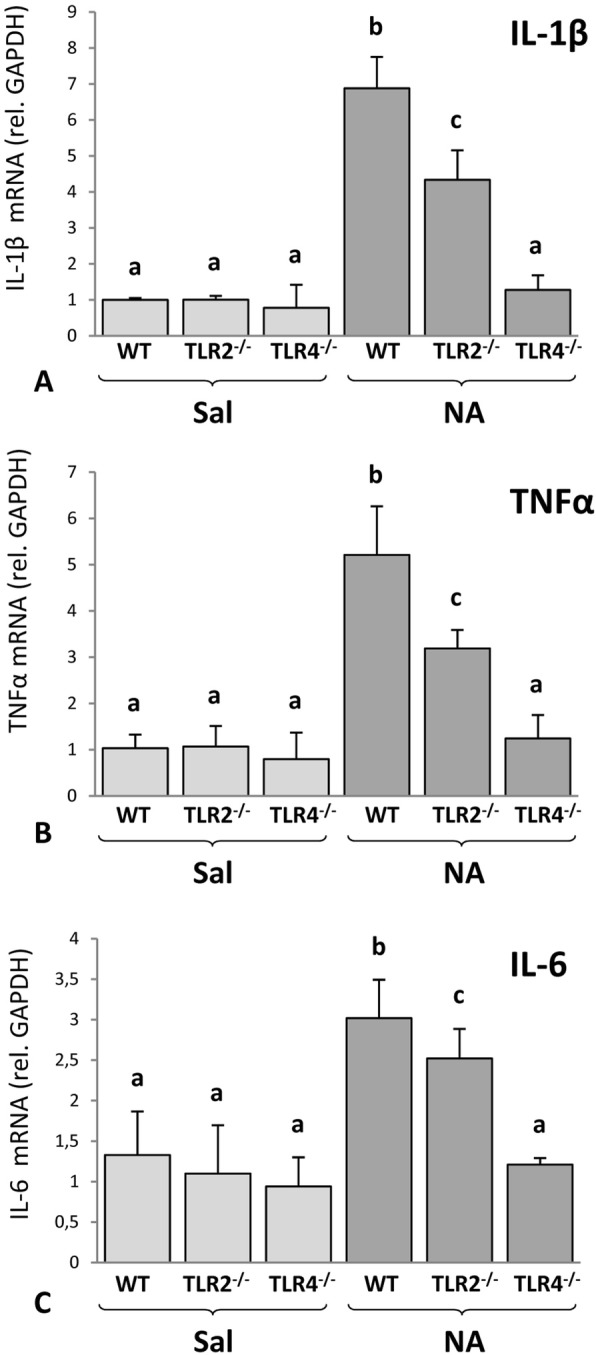
Pro-inflammatory cytokines expression in the hypothalamus of TLR2- and TLR4-deficient mice after NA injection. The mRNA levels of IL-1β (**a**), TNFα (**b**), and IL-6 (**c**) was measured by qPCR in hypothalamic tissue from WT, TLR2^-/-^, and TLR4^-/-^ mice obtained 6 h after the ICV injection of saline or NA. mRNA levels of the target genes were expressed relative to the expression level of GAPDH using the Pfaffl method. The injection of NA induced the overexpression of the three cytokines in WT mice and in TLR2^-/-^ mice, the increase being milder in the latter strain. However, in TLR4^-/-^ mice injected with NA, the expression levels of the three cytokines were similar to those on saline controls. The histograms show means + SD of *n* = 3 saline injected and *n* = 5 NA-injected animals of each strain. Two-way ANOVA was used to compare the means of the groups. Letters (a–c) on top of the bars indicate the absence (same letter) or presence (different letter) of a significant difference (*P* < 0.05) between groups

The NA-induced inflammatory reaction was further analyzed by quantifying the mRNA levels of pro-inflammatory cytokines by qPCR. Wild type, TLR2^-/-^, and TLR4^-/-^ mice were saline or NA injected and sacrificed 6 h later. The hypothalamus was dissected out for total RNA isolation and qPCR. This brain region was preferred for this study because it is surrounding the third ventricle, the route of the cerebrospinal fluid flow just after the injection of NA within the lateral ventricle, and also distant enough from the injection site as to be devoid of injection damage. NA induced a significant increase of IL-1β expression (Fig. 3a) in the hypothalamus of WT and TLR2^-/-^ mice compared to saline-injected animals (*P* < 0.001 and *P* < 0.005 respectively), but not in TLR4^-/-^ mice. Similarly, TNFα levels (Fig. 3b) were higher after NA-injection in WT (*P* < 0.001 vs saline WT) and in TLR2^-/-^ (*P* < 0.005 vs saline TLR2^-/-^) mice, but not in TLR4^-/-^ animals. Interleukin-6 (IL-6) mRNA levels behaved in a similar way (Fig. 3c), presenting increased values in WT (*P* < 0.001 vs saline WT) and in TLR2^-/-^ mice (*P* < 0.005 vs saline TLR2^-/-^) injected with NA, but not in TLR4^-/-^ animals. Therefore, in TLR4^-/-^ animals, no differences were observed between NA-treated and saline-injected animals for any of the cytokines analyzed. Also, when comparing WT and TLR2^-/-^ strains both under NA stimulation, the overexpression of IL-1β, TNFα, and IL-6 was milder in TLR2^-/-^ mice than in WT ones (*P* < 0.05 vs NA WT for the three cytokines).

### In cultured microglia, NA induced a TLR4-dependent overexpression of pro-inflammatory cytokines

**Fig. 4:**
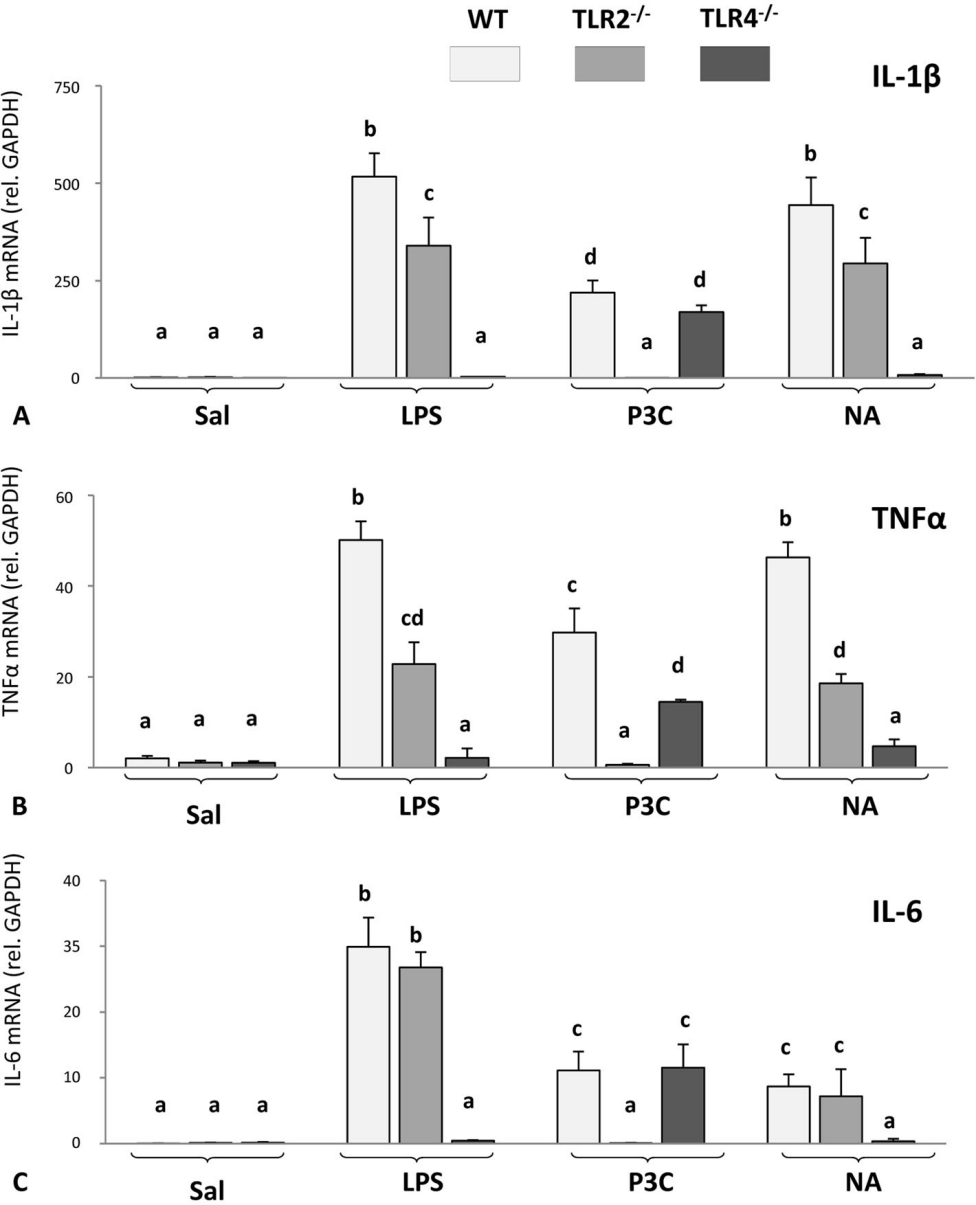
Pro-inflammatory cytokines in cultured microglia from TLR2^-/-^ and TLR4^-/-^ mice after in vitro NA stimulation. Microglia cultures isolated from WT, TLR2^-/-^, and TLR4^-/-^ mice strains were stimulated in vitro with LPS (a TLR4 agonist), the synthetic lipoprotein P3C (a TLR2 agonist), or neuraminidase (NA) for 6 h. Total RNA was then isolated, and the mRNA levels of IL-1β (**a**), TNFα (**b**), and IL-6 (**c**) were quantified by qPCR. mRNA levels are expressed relative to GAPDH using the Pfaffl method. While NA was able to stimulate cytokine production in WT and TLR2^-/-^ microglial cells, it was unable to do so in TLR4^-/-^ cells. As expected, and similarly to NA, LPS induced cytokine production in WT and TLR2^-/-^ microglia, but not in TLR4^-/-^ cells. On the contrary, and also as expected, P3C was able to induce cytokine overexpression in WT and TLR4^-/-^ microglia, but not in TLR2^-/-^ cells. The histograms show means + SD of *n* = 3–5 independent cultures. Two-way ANOVA was used to compare treatments. Letters (**a**–**c**) above each bar indicate the absence (same letter) or presence (different letters) of a statistically significant difference (*P* < 0.001) between the compared groups

To find out whether microglial cells can initiate by themselves the inflammatory response provoked by NA, and also to elucidate if NA acts through TLR2 and/or TLR4 receptors, microglia were isolated from WT, TLR2^-/-^, and TLR4^-/-^ mice to obtain cultures of microglial cells. These primary microglial cultures were stimulated with NA or with specific ligands for TLR2 and TLR4: P3C, a synthetic triacylated lipoprotein which specifically activates TLR2, and ultrapure LPS, which is a specific ligand for TLR4. Control cultures were added saline. Six hours after NA, P3C, or LPS addition, total RNA was isolated and the expression of the pro-inflammatory cytokines IL-1β, TNFα, and IL-6 was quantified by qPCR (Fig. 4).

Two-way ANOVA pointed out significant differences in the expression levels of IL-1β (*F* = 5.9; *df* = 11, 32; *P* < 0.001), TNFα (*F* = 4.7; *df* = 11, 32; *P* < 0.001), and IL-6 (*F* = 12.8; *df* = 11, 32; *P* < 0.001) between different treatments. The expression of the three pro-inflammatory cytokines in WT microglia cultures increased after treatment with LPS, P3C, and NA compared to saline-treated cultures (Fig. 4). This result demonstrates that, along with LPS and P3C, NA is also able to directly activate microglial cells.

Microglia isolated from TLR2^-/-^ responded with an increased expression of IL-1β (Fig. 4a), TNFα (Fig. 4b), and IL-6 (Fig. 4c) after LPS and NA treatments but, as expected, no response was observed after treatment with the TLR2 agonist P3C.

Conversely, TLR4^-/-^ microglial cells did not show an increase in the expression of any cytokine after LPS stimulation, as was expected since LPS is a TLR4 agonist (Fig. 4). Interestingly, and similarly to LPS, NA was not able either to induce a response in TLR4^-/-^ microglial cells. It is noteworthy to mention that TLR4^-/-^ microglia was activated by the TLR2 agonist P3C, indicating that microglial cells are capable of initiating an immune response independent of TLR4. Of note, the IL-1β and TNFα responses of TLR2^-/-^ microglia to LPS were not as that of WT microglia, but slightly milder. Because the dose of LPS used was relatively high, in this experimental condition, WT microglia could have been activated not only by LPS binding to TLR4 but additionally by contaminants present in the LPS preparations acting through other receptors. This could explain the higher activation level of WT microglia compared to TLR2^-/-^ microglia. A similar explanation could be applied to the lower TNFα response of TLR4^-/-^ microglia to P3C, as this agonist was also used at a relatively high dose.

Therefore, these results point that NA can directly activate microglial cells, probably acting through the receptor TLR4.

### Inactivation or inhibition of the sialidase activity of NA abrogate its capacity of activating microglial cells

The above results demonstrate that NA per se is able to activate microglial cells. However, the question remains if this happens thanks to its recognition as a PAMP by pattern recognition receptors of the host, or if its sialidase activity is involved in the microglial cell activation process. To address this issue, cultures of WT microglial cells were exposed to native NA, heat-inactivated NA, and native NA with sialidase inhibitors, namely oseltamivir phosphate and NADNA. Microglial cultures were exposed to these conditions for 24 h and then processed for qPCR quantification of IL-1β and TNFα expression.

**Fig. 5:**
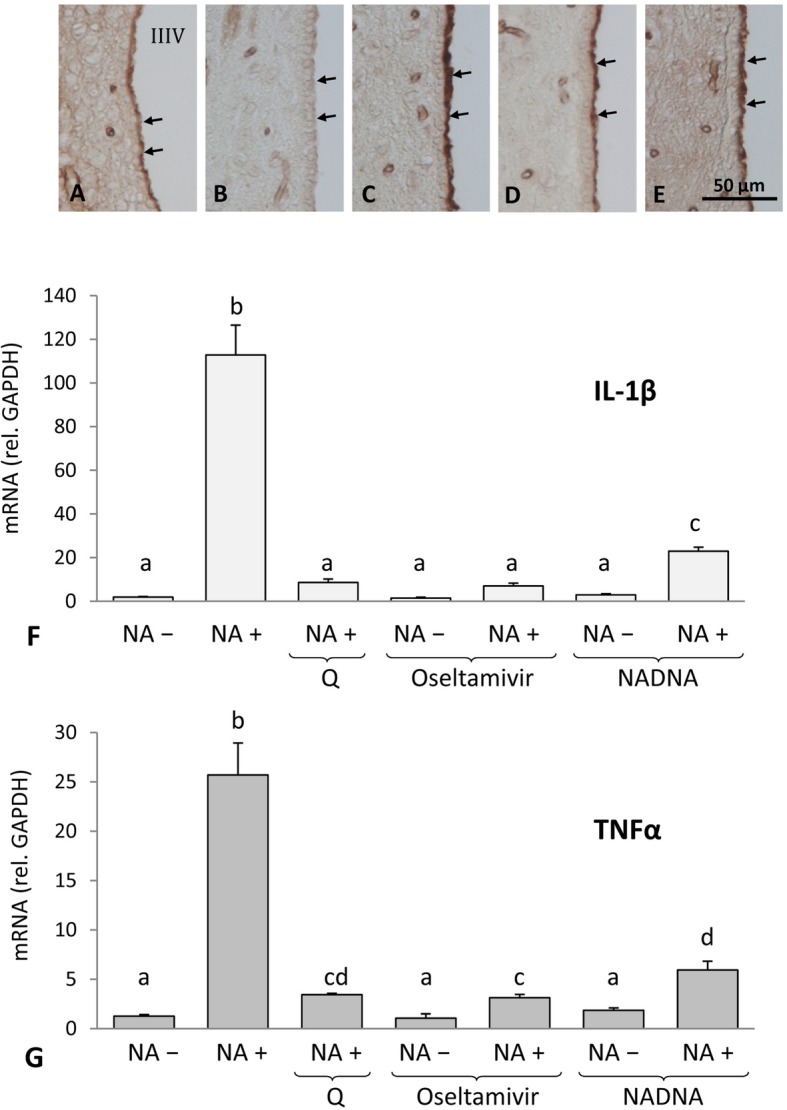
Effect of NA inactivation/inhibition on microglial response. Lectin histochemistry (**a**–**e**) was used to demonstrate the inactivation of the sialidase activity of NA. Tissue sections containing the hypothalamus ventricular wall were incubated with diluent solution (**a**), with NA (**b**), with heat-inactivated NA (**c**), with NA and oseltamivir phosphate (**d**), or with NA and NADNA (**e**). SNA lectin (with sialic acid affinity) binding on the ventricular surface reveals the sialidase activity (SNA-negative) or its inactivation/inhibition (SNA-positive). Microglia cultures from WT mice were exposed during 24 h to heat-inactivated NA (Q) or to native NA in the presence of the sialidase inhibitors oseltamivir phosphate and NADNA (**f**, **g**). The activation of microglial cells was monitored by measuring the overexpression of IL-1β (**f**) and TNFα (**g**) by qPCR. Native NA induced the expression of both cytokines, but was unable to do so after heat-inactivation. Furthermore, oseltamivir and NADNA also prevented the stimulation effect of native NA on microglia. The histograms show means + SD of *n* = 7 independent cultures. One-way ANOVA was used to compare treatments. Letters (**a**, **b**) above each bar indicate the absence (same letter) or presence (different letters) of a statistically significant difference (*P* < 0.05) between the compared groups

Prior to the experiment, the efficiency of the heat inactivation and the effective dose of the inhibitors were checked by lectin histochemistry, using a sialic acid–specific lectin such as SNA (Fig. 5a–e). This test was performed on brain sections containing ependyma, whose surface is rich in sialic acid. While the ependymal surface was SNA-positive in non-treated sections (Fig. 5a), it turned SNA-negative after incubation of the sections with NA (Fig. 5b), indicating the removal of sialic acid by the sialidase activity of NA. Heat inactivation of NA (Fig. 5c), or the presence of oseltamivir (Fig. 5d) or NADNA (Fig. 5e), abolished the sialidase activity of NA, as demonstrated by the binding of SNA to the ependymal surface.

As expected, native NA was able to stimulate the overexpression of both IL-1β (Fig. 5f) and TNFα (Fig. 5g) in cultured microglia (*P* < 0.001 vs control without NA, for both genes). However, such capacity was greatly diminished after heat inactivation of NA, as well as when adding oseltamivir or NADNA along with native NA to the culture (Fig. 5f, g). Still, a marginal activation of microglia by these inactive/inhibited forms of NA could be observed (compare NA + Q, NA + oseltamivir, and NA + NADNA with any NA–condition).

Therefore, these results indicate that it is the sialidase activity of NA, and not so much the NA protein itself, that induces the activation of microglia.

## Discussion

We previously reported that the injection of NA from *Clostridium perfringens* into the lateral ventricle of rats provokes an acute inflammatory process, characterized by the activation of astrocytes and microglia, and the recruitment of lymphocytes and monocytes. The rationale of using NA was based on the fact that various pathogens, some of which are able to invade the CNS, bear this enzyme in its structure. These include viruses like mumps virus or influenza virus, and bacteria like *Streptococcus pneumoniae*, a causative agent of meningitis. Thus, the sole presence of NA in the ventricular cavities triggers a neuroinflammatory response and represents an experimental model of sterile inflammation. However, the mechanism by which NA is able to induce such inflammation is unknown.

Here we have shown that mice deficient in the pattern recognition receptor TLR2 develop a slightly milder inflammatory response after ICV administration of NA, while in mice deficient in TLR4, such response is greatly (although not completely) abolished. Experiments using highly enriched microglia cultures from TLR2^-/-^ and TLR4^-/-^ strains pointed that the TLR4 receptor of microglial cells is essential for NA-induced microglial activation, while the TLR2 receptor plays a partial role. Besides, NA-induced microglial activation was almost completely dependent on the sialidase activity of the enzyme, as demonstrated by its heat-inactivation and by the use of sialidase inhibitors. This highlights the importance of terminal sialic acid dynamics and neuramidases in the regulation of the immune response.

It is well known that TLR receptors play a critical role in immune response [3]. They do so by recognizing molecular patterns arising from either invading pathogens or the host itself. Our results demonstrate that bacterial NA also acts through TLR receptors, specifically TLR2 and TLR4, to induce the activation of the innate immune response within the CNS. Although such response was almost completely abolished in TLR4^-/-^ mice, some activation still occurred, as microglial cells appeared partially reactive in IBA1 immunostained tissue sections (Fig. 1i, l), and a slight increase in IL-1β-positive cells could be observed (Fig. 2g, h). This could be explained by the involvement of other pattern recognition receptors, such as TLR2. Evidences supporting this possibility were obtained herein, as the TLR2^-/-^ strain presented a reduced cytokine response to NA compared to WT mice, both in vivo (Fig. 2) and in vitro (Fig. 3). In any case, the results presented here demonstrate that the receptor TLR4 plays a crucial role in NA-induced neuroinflammation.

On the other hand, previous studies showed that NA also induces the overexpression of the adhesion molecule ICAM-1 in endothelial cells [29]. ICAM-1 is relevant for the recruitment of lymphocytes during inflammation. The ICV injection of NA in TLR2^-/-^ and TLR4^-/-^ mice produced an increase in the expression of ICAM-1 similar to that of WT mice (Additional file 1). Therefore, the diminished inflammatory response in both mutant strains is specific of TLR2 and TLR4 receptors respectively, like other aspects of the inflammatory process (e.g., ICAM-1 overexpression) remained unaltered.

Both TLR2 and TLR4 have been frequently reported to participate in various inflammatory processes developing within the CNS. TLR2 is involved in the sterile inflammation occurring in cerebral ischemia [37, 38]. Also, in bacterial meningitis provoked by *Streptococcus* (which bears NA), TLR2 activation mediates neuronal death [39] and microglial apoptosis [40]. On the other hand, although bacterial LPS is the stereotype ligand of TLR4, this receptor also has been shown to be essential in the sterile inflammation occurring both after hemorrhagic stroke [41, 42] and in ischemic stroke [43].

The different TLR family members may interact and form dimers in the cellular surface, which generates an additional source of variability in the cellular response to ligand binding [44]. In pneumococcal meningitis, the interaction of TLR2 and TLR4 is required for the immunologic reaction [45]. Similarly, in the inflammatory process that follows a traumatic brain injury, TLR2/TLR4 heterodimers are activated by the hemoglobin released [46]. The possibility that NA may require the dimerization of TLR2 and TLR4 for microglial activation remains an open question, as both receptors were necessary, although to different extent, for microglial activation by NA.

A significant feature of microglial activation by bacterial NA found here was the requirement of an intact sialidase activity of the enzyme. The suppression of such activity by heat-inactivation or by specific sialidase inhibitors resulted in the inability of NA to activate microglial cells (Fig. 5). Interestingly, the NA protein itself seemed not to be recognized as a PAMP to trigger microglial activation, as demonstrated by the unresponsiveness of microglia cultures exposed to heat-inactivated NA. However, subtle microglial activation was observed when using heat-inactivated or inhibited NA, which could be explained by the recognition of NA as a PAMP. However, an incomplete inactivation of the sialidase activity might also explain this result.

It is possible that both, the molecular pattern of the NA protein and the sialidase activity, are required for pattern receptor activation. In fact, it has been recently revealed an intriguing mechanism of TLR activation where the binding of a ligand triggers an initial cellular response that elicits the translocation of the endogenous sialidase Neu1 from its lysosomal location to the cell surface, to later desialylate the receptor, thus allowing the full activation of the signaling cascade. Once translocated to the cell surface, Neu1 sialidase forms a complex with TLR-2, -3, and -4 receptors, which are highly glycosylated, and removes α-2,3-sialyl residues. In macrophages and dendritic cells, LPS-induced NF-κB activation was dependent on the removal of α-2,3-sialyl residues present in TLR4 by Neu1. Once desialylated, TLR4 could dimerize and form a complex with other components (as myeloid differentiation primary response 88, MyD88) of its signaling cascade [47, 48]. In this mechanism, the matrix metalloproteinase-9 (MMP9) is required for Neu1 activation [49].

Neu1 inhibition with oseltamivir phosphate prevented LPS-induce TLR4 activation and the subsequent production of pro-inflammatory cytokines in macrophages [47]. In our microglial cultures treated with this inhibitor there was no cytokine response, which could be a consequence of the inhibition of both the exogenous NA and the endogenous Neu1. However, when heat-inactivated NA was used (in the absence of oseltamivir and with intact Neu1 activity) no cytokine response occurred either. These results suggest that (i) the sole presence of an exogenous sialidase (e.g., that of pathogens) is sufficient to trigger microglial cytokine response, being the endogenous Neu1 not essential, and (ii) inactive NA is not recognized as a PAMP, so probably Neu1 is not translocated to the cell surface to initiate the signaling cascade. Regarding the requirement for ligand binding to the TLR receptor (in our scenario, TLR4 and/or TLR2), the proper NA protein could act as a PAMP itself and, along with TLR desialylation by NA, would facilitate TLR signaling activation. Alternatively, the sole desialylation of the receptor would be sufficient to trigger its activation. None of these possibilities can be confirmed with the present results.

Another player in the above-explained mechanisms has been recently uncovered. Siglecs are another type of pattern recognition receptors with affinity for sugar residues. A broad interaction of various Siglecs with TLRs occurs at the cellular surface, and it has been shown that they provide a constitutive blockade for TLRs activation [50]. Specifically, murine Siglec-E strongly binds to TLR4, and desialylation of TLR4 by Neu1 releases such interaction. In our experimental setting, microbial NA could act in a similar manner to promote Siglec E/TLR4 decoupling and TLR4 activation. Recently, Siglec-H has been described as specific for microglial cells, not present in CNS-associated macrophages or in CNS-infiltrating monocytes [51].

Therefore, Siglecs pose a restriction to TLR activation, which is released after desialylation by Neu1 or, eventually, NA. In this way, TLR activation seems to occur in two phases: an initial mild activation triggered by ligand binding to the TLR receptor, followed by the translocation of Neu1 to the cell surface to desialylate the receptor a release its blockade by Siglecs, what in turn would allow a full cellular inflammatory response. When a microbial sialidase is present, Neu1 would not be essential for such cellular response.

Thus, Neu1 plays a pivotal role in immune cells activation and emerges as a promising target in inflammatory processes. In fact, *Neu1* deletion provided resistance to endotoxemia, and sialidase inhibitors were able to protect mice against it [50]. Microbial sialidases worsen the symptoms, as they exacerbate sepsis by a similar mechanism of Siglec release; the treatment with bacterial sialidase inhibitors also improves the outcome of this type of infection [52]. Therefore, a strategy of sialidase inhibition may help in controlling inflammation, but the specificity of the inhibitor must be taken into account, as exogenous and endogenous sialidases may be differently susceptible.

It has been shown that bacterial NA favors the formation of biofilms, which promotes tissue colonization [53]. Also, viral NA is relevant for the dispersion of new virions, a process extensively studied for influenza virus, in which NA determines its virulence [54, 55]. Interestingly, although influenza virus targets the respiratory system, it may also cause neurologic complications [22–24]. Cases of schizophrenia and autism have been related to influenza infections during pregnancy [56], as well as encephalopathies or encephalitis [23, 57, 58]. As NA is a common factor in various microbe-provoked CNS pathologies, finding out the role of NA and its mechanism of action is of great interest in treating those diseases.

## Conclusions

The results exposed here demonstrate that i) NA induces inflammation by acting mainly through TLR4 receptor and secondarily through TLR2; ii) microglial cells, which express TLR4, are a target of NA and a relevant player in such inflammatory process; and iii) the sialidase activity of NA, more than the NA protein itself, induces TLR4 signaling. The inhibition of the sialidase activity of NA is one of the most extensively used antiviral strategies aimed to prevent the dispersion of virions from infected host cells [26, 59, 60]. Sialidase inhibition also ameliorated bacterial sepsis [52]. The results presented here highlight a novel effect of NA inhibitors, which is the blockade of TLR4 signaling, the consequences of which should be taken into consideration in the future.

## Supplementary information

**Additional file 1: **ICAM-1 immunostaining after NA injection in WT, TLR2^-/-^, and TLR4^-/-^ mice. Images taken from the septofimbria (A–C) and the hypothalamus (D–F) of WT (A, D), TLR2^-/-^ (B, E), and TLR4^-/-^ (C, F) mice, 24 h after the injection of NA. The ICAM-1 label in samples from saline-treated animals was almost undetectable (not shown). Quantification of ICAM-1 positive cells was carried out in the septofimbria (G) and the hypothalamus (H). Bars are the mean + SD of *n* = 3 saline injected or *n* = 9–10 NA-injected animals (for each strain). Kruskal-Wallis test was used for means comparisons. Letters a and b on top of the bars indicate the absence (if same letter) or presence (if different letter) of a significant difference between the groups compared (*P* < 0.001). LV = lateral ventricle; SF = septofimbria; IIIV = third ventricle

## Data Availability

There is no new software, databases, and application/tool available, apart from the reported in the present article.

## Abbreviations

ABC: Avidin-biotin-complex
CNS: Central nervous system
DAMPs: Damage-associated molecular patterns
DMEM-F12: Dulbecco’s Modified Eagle Medium/Nutrient Mixture F-12
EDTA: Ethylenediaminetetraacetic acid
FBS: Fetal bovine serum
GAPDH: Glyceraldehyde 3-phosphate dehydrogenase
IBA1: Ionized calcium-binding adaptor molecule 1
ICV: Intracerebroventricular
IFNβ: Interferon beta
IL-1β: Interleukin 1 beta
IL-6: Interleukin 6
LPS: Lipopolysaccharide
MMP9: Matrix metalloproteinase-9
MyD88: Myeloid differentiation primary response 88
NA: Neuraminidase
NADNA: N-acetyl-2,3-dehydro-2-deoxyneuraminic acid
NF-κB: Nuclear factor Kappa B
qPCR: Quantitative PCR
PAMPs: Pathogen-associated molecular patterns
P3C: Synthetic triacylated lipoprotein Pam3CSK4
PBS: Phosphate-buffered saline
ROS: Reactive oxygen species
SNA: *Sambucus nigra* agglutinin
TLR2: Toll-like receptor 2
TLR4: Toll-like receptor 4
TLR2^-/-^: Toll-like receptor 2 null mutant mice
TLR4^-/-^: Toll-like receptor 4 null mutant mice
TNFα: Tumor necrosis factor alpha
WT: Wild type
