# Supplementary figures and images for: Microglial activation by microbial neuraminidase through TLR2 and TLR4 receptors
Source: J Neuroinflammation. 2019 Dec 2;16:245. doi: 10.1186/s12974-019-1643-9 (PMC6889729; doi:10.1186/s12974-019-1643-9)

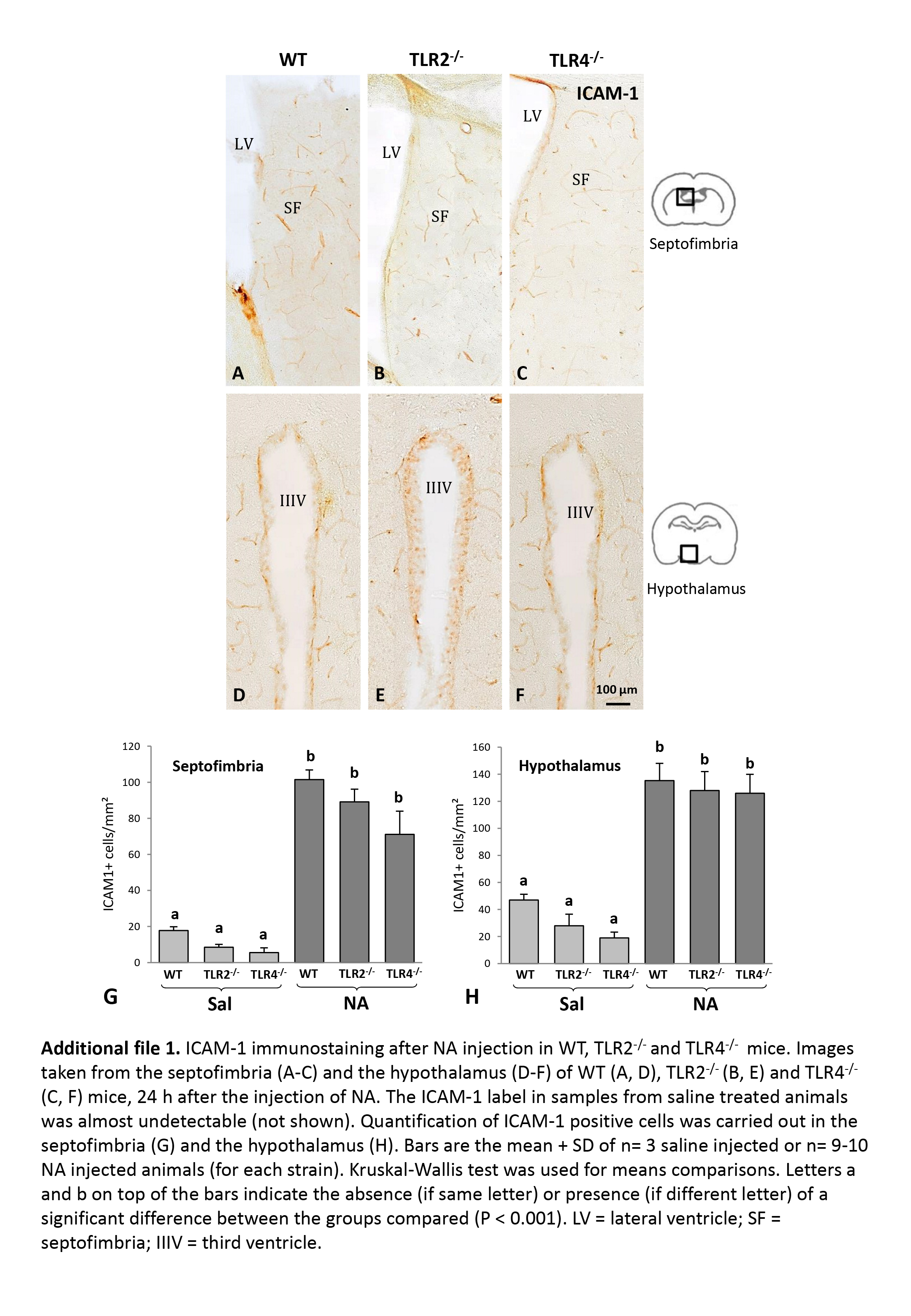

Supplement: Supplementary file 1 — Additional file 1: ICAM-1 immunostaining after NA injection in WT, TLR2-/-, and TLR4-/- mice. Images taken from the septofimbria (A–C) and the hypothalamus (D–F) of WT (A, D), TLR2-/- (B, E), and TLR4-/- (C, F) mice, 24 h after the injection of NA. The ICAM-1 label in samples from saline-treated animals was almost undetectable (not shown). Quantification of ICAM-1 positive cells was carried out in the septofimbria (G) and the hypothalamus (H). Bars are the mean + SD of n = 3 saline injected or n = 9–10 NA-injected animals (for each strain). Kruskal-Wallis test was used for means comparisons. Letters a and b on top of the bars indicate the absence (if same letter) or presence (if different letter) of a significant difference between the groups compared (P < 0.001). LV = lateral ventricle; SF = septofimbria; IIIV = third ventricle [file 12974_2019_1643_MOESM1_ESM.jpg]
